# Network analysis of polymicrobial chronic wound infections in Masanga, Sierra Leone

**DOI:** 10.1186/s12879-023-08204-0

**Published:** 2023-04-18

**Authors:** Sarah Sandmann, Jonathan Vas Nunes, Martin P. Grobusch, Maxwell Sesay, Martin A. Kriegel, Julian Varghese, Frieder Schaumburg

**Affiliations:** 1 Institute of Medical Informatics, University of Münster, Münster, Germany; 2 Masanga Medical Research Unit (MMRU), Masanga, Sierra Leone; 3 Department of Infectious Diseases, Center of Tropical Medicine and Travel Medicine, Amsterdam University Medical Centers, Amsterdam, The Netherlands; 4 Section of Rheumatology and Clinical Immunology, Department of Medicine, University Hospital Münster, Münster, Germany; 5 Department of Translational Rheumatology and Immunology, Institute of Musculoskeletal Medicine, University of Münster, Münster, Germany; 6 Cells in Motion Interfaculty Centre, University of Münster, Münster, Germany; 7 Department of Immunobiology, Yale University School of Medicine, New Haven, USA; 8 Institute of Medical Microbiology, University of Münster, Münster, Germany

**Keywords:** Microbiome, Wound infection, Africa, Community networks, *Staphylococcus aureus*, *Pseudomonas aeruginosa*, Panton-Valentine leukocidin

## Abstract

**Background:**

Chronic wounds are frequently colonized or infected with multiple bacterial or fungal species, which can both promote or inhibit each other. Network analyses are helpful to understand the interplay of these species in polymicrobial infections. Our aim was to analyse the network of bacterial and fungal species in chronic wounds.

**Methods:**

Swabs (n = 163) from chronic wound infections (Masanga, Sierra Leone, 2019–2020) were screened for bacterial and fungal species using non-selective agars. Some of these wounds were suspected but not confirmed Buruli ulcer. Species identification was done with MALDI-TOF mass spectrometry. Network analysis was performed to investigate co-occurrence of different species within one patient. All species with n ≥ 10 isolates were taken into account.

**Results:**

Of the 163 patients, 156 had a positive wound culture (median of three different species per patient; range 1–7). *Pseudomonas aeruginosa* (n = 75) was the dominating species with frequent co-detections of *Klebsiella pneumoniae* (21 cases; OR = 1.36, 95%CI: 0.63–2.96, p = 0.47), *Staphylococcus aureus* (14 cases; OR = 1.06, 95%CI: 0.44–2.55, p = 1) and *Proteus mirabilis* (13 cases; OR = 0.84, 95%CI: 0.35–1.99, p = 0.69).

**Conclusion:**

The culturome of chronic wounds in Sierra Leonean patients is highly diverse and characterized by the co-occurrence of *P. aeruginosa*, *K. pneumoniae* and *S. aureus*.

**Supplementary Information:**

The online version contains supplementary material available at 10.1186/s12879-023-08204-0.

## Background

Chronic wounds are common in underserved populations with limited access to healthcare and are frequently colonized or infected with polymicrobial bacterial communities. The composition of the wound microbiome is of clinical relevance as the presence of certain species (e.g. *Enterobacter*) or a stable microbiota community in diabetic foot ulcera were predictive for delayed healing [1, 2].

The interaction of multiple species within the wound is complex as they affect each other by the secretion of molecules. These molecules can alter the tolerance to antibiotics, virulence or biofilm formation as shown for *Pseudomonas aeruginosa* and *Staphylococcus aureus* [3]. The *S. aureus* protein toxin Panton-Valentine leukocidin (PVL) is of particular interest in resource limited settings as it is associated with severe skin and soft tissue infections (SSTI) and much more common among *S. aureus* from sub-Saharan Africa (up to 74%) compared to Europe (1.4%) [4–6]. One in vitro study suggests that PVL could be a competitive advantage for *S. aureus* in the interplay with *P. aeruginosa*, but this finding has not been yet confirmed in vivo [7].

In the past years, network-based analytical approaches were developed to study the structure and interaction of polymicrobial communities [8]. These network analyses make use of mathematical modelling to identify patterns within bacterial communities. While first results on microbial networks are available from chronic wounds in developed countries, this information is absent for resource-limited settings. Results from high-income countries should not be extrapolated to low- and middle-income countries as they differ in host-related factors (e.g. malnutrition, HIV-infection), pathogens (e.g. *Mycobacterium ulcerans*, *Blastomyces*, *Coccidioides*), microbiota, and the environment (e.g. humidity, sanitary system, abundance of flies) [9–11]. We therefore performed a network analysis (i) to characterize the bacterial community of chronic wounds in patients from Sierra Leone and (ii) to test if PVL-positive *S. aureus* is associated with the absence of specific species (e.g. *P. aeruginosa*).

## Methods

### Study population

The study made use of an already existing database of bacterial species from chronic wounds in patients (n = 163) from Masanga, Sierra Leone (July 2019–November 2020) [12]. These wounds showed characteristics of Buruli ulcer and could be secondary infections to *Mycobacterium ulcerans* [12].

In brief, patients with any kind of wounds (e.g. wound originating from trauma, infection, burn or drug reaction) and an informed consent to participate were included. If a person was not of legal age, the guardian gave the informed consent. Patients with closed wounds (e.g. from blunt trauma, haematomas) and surgical site infections were excluded.

### Microbiology

After cleaning the wound from bandages, traditional leaves or necrotic skin with a sterile cotton gauze, one swab (Transswab, MWE, Corsham, England) per patient was taken applying slight pressure from both the (undermined) edges and the central areas of the wound (Essen Rotary technique) [13]. Samples were stored in Amies transport medium at 2–7 °C until shipment to Germany (median time between sampling and culture: 3.8 months). Details on the culture conditions are described elsewhere [12]. MALDI-TOF mass spectrometry (Microflex Bruker, Bremen, Germany) and the MBT Compass software (version 4.1.80, Bruker) were used for species identification. All *S. aureus* isolates were screened for PVL using a commercial test kit (eazyplex® MRSAplus, Amplex, Gars-Bahnhof, Germany).

### Network analysis

Network analysis was performed using R 4.2.1 [14]. The R package “igraph” was used for visualization [15]. Interaction networks were generated adapting an example published by Varghese et al., analysing co-occurring and persistent symptoms in COVID-19 [16]. In brief, every species is represented by a node. The size of each node is correlated with the number of observations (precise numbers are additionally reported). Intersects, i.e. co-occurring species within one patient, were visualized by edges. The thickness of an edge corresponds to the number of observations; precise numbers are additionally reported for intersects ≥ 10. A missing edge between two nodes indicates that co-occurrence was never observed for these two species. To reduce the complexity of the network and improve visualization, only species detected in ≥ 10 patients were considered.

Heatmaps, visualizing all species detected and their co-occurrence, were generated using R 4.2.1 and R package “pheatmap” [17]. For *S. aureus*, we differentiate between PVL-positive (PVL +) and PVL-negative (PVL -) isolates.

### Cross-streak assay

To assess the interaction of *S. aureus* and *P. aeruginosa*, we performed a cross-streak assay with overnight cultures [7, 18]. One loop tip (ca. 1 µl) of *P. aeruginosa* was horizontally streaked on a Columbia blood agar plate. An identical amount of one *S. aureus* colony was streaked vertically crossing the *P. aeruginosa* streak in the middle of the plate. After incubation (18–24 h, ambient air, 35 ± 1 °C), the growth of *S. aureus* was scored (0–2 points: 0 = full inhibition by *P. aeruginosa*, 1 = interruption of the *S. aureus* streak at the crossing with the *P. aeruginosa* streak, 2 = no interruption of the *S. aureus* streak). The scores of the independent tests were added to quantify the *S. aureus* growth performance (*S. aureus* growth score).

### Statistics

The diversity indices (Shannon, Simpson) were calculated with “R” as implemented in the package “abdiv”. Statistical testing, applying Fisher’s Exact Test, was performed using R and the base function fisher.test (alternative: two.sided). The *S. aureus* growth scores were compared with the Wilcoxon rank sum test with continuity correction.

## Results

Of the 163 eligible patients (11% females, median age of 40 years, range: 0–88), 156 had a positive wound culture and were entered into the final analysis. The majority of wounds were located at the lower limb (85.9%, n = 140) and had a median diameter of 10 cm. Wounds were superficial (84%, n = 137), 53% of them had deep edges (n = 86) [12]. Results from antimicrobial susceptibility testing of these isolates was already reported elsewhere [12].

Three different species per patient (median, range 1–7) were detected, with a total number of 60 different species and 461 isolates in the whole dataset. The most common species were *P. aeruginosa* (n = 75/461, 16.3%), *Klebsiella pneumoniae* (n = 42/461, 9.1%), *Proteus mirabilis* (n = 31/461, 6.7%) and *S. aureus* (n = 30/461, 6.5%). Of all *S. aureus*, 43% (n = 13) were methicillin-resistant (*mecA* positive).

The only fungi were *Candida tropicalis* (n = 6/461, 1.3%), *Candida orthopsilosis* (n = 2/461, 0.4%) and *Candida krusei* (n = 1/461, 0.2%). Anaerobes were rarely detected: *Bacteroides fragilis* (n = 4/461, 0.9%) and *Bacteroides thetaiotaomicron* (n = 2/461, 0.4%). All wounds together had a Shannon index of 3.29 and a Simpson index of 0.94.

**Fig. 1:**
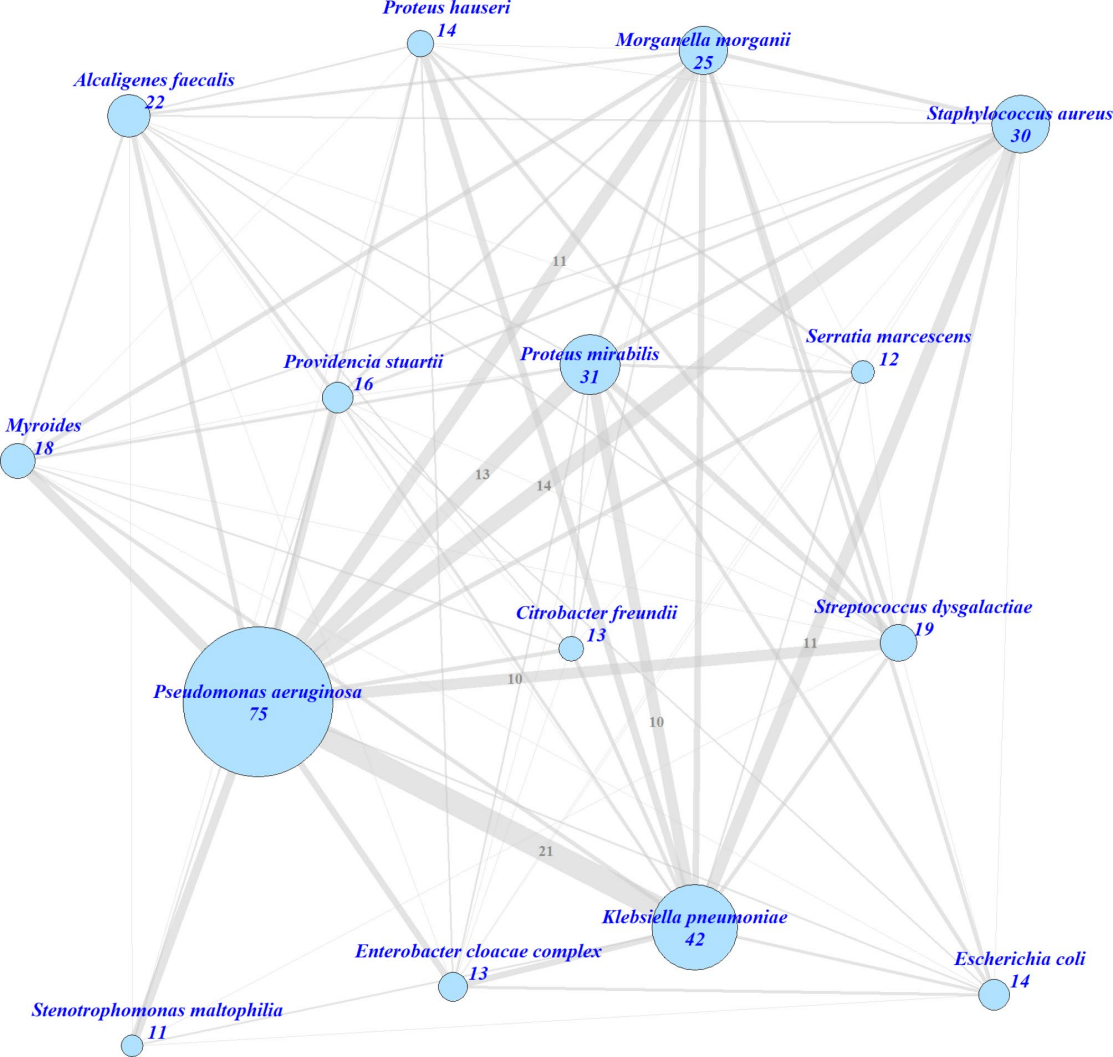
Network analyses of bacteria from chronic wound infections, Sierra Leone. Only species with a total number of ≥ 10 isolates were included. Node size and node number correspond to the number of isolates of the respective species. Thickness of grey edges corresponds to the co-occurrence of the displayed species

We performed a network analysis to identify those species that frequently co-occur in individual wounds (Fig. 1). *P. aeruginosa* was the dominating species with frequent co-detections of *K. pneumoniae* (21 cases; OR = 1.36, 95%CI: 0.63–2.96, p = 0.47), *S. aureus* (14 cases; OR = 1.06, 95%CI: 0.44–2.55, p = 1) and *P. mirabilis* (13 cases; OR = 0.84, 95%CI: 0.35–1.99, p = 0.69). Although the network analysis suggests a triangle pattern of *P. aeruginosa*, *K. pneumoniae* and *S. aureus* (Fig. 1), the analysis of co-occurrence in individual patients reveals that only six patients carried all three species out of 30 being colonized with *S. aureus* (Supplementary Fig. 1).

The PVL protein toxin was detected in 23% (n = 7/30) of all *S. aureus* isolates.

We tested our hypothesis that the presence of PVL-positive *S. aureus* is associated with the presence of certain species. We did not find any significant associations for *P. aeruginosa* (OR = 0.38, 95%CI: 0.03–2.93, p = 0.40), *P. mirabilis* (OR = 2.57, 95%CI: 0.17–29.79, p = 0.57), *S. dysgalactiae* (OR = 0, 95%CI: 0–3.65, p = 0.30) or *K. pneumoniae* (OR = 0.63, 95%CI: 0.05–4.99, p = 1).

**Fig. 2:**
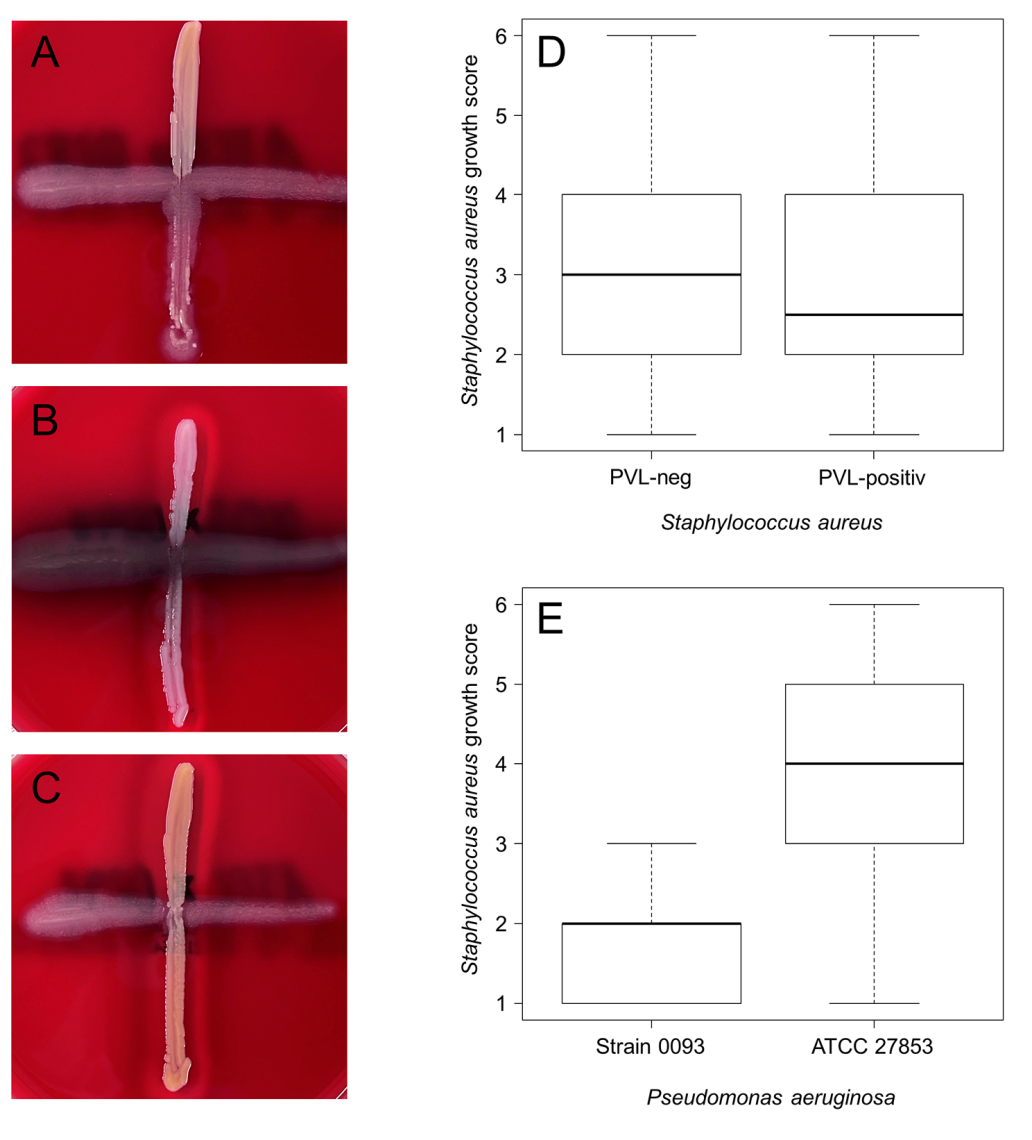
Cross-streak assay with *Pseudomonas aeruginosa* and *Staphylococcus aureus*. Two different *P. aeruginosa* strains (0093, ATCC 27,853, horizontal) were cross-streaked with seven PVL- positive and seven PVL-negative *S. aureus* from this study (A-C). The growth of *S. aureus* was scored 0 points (full inhibition by *P. aeruginosa*, A), 1 point (interruption of the *S. aureus* streak at the crossing with the *P. aeruginosa* streak, B) and 2 points (no interruption of the *S. aureus* streak, C). The scores of three independent tests were added to calculate the *S. aureus* growth score (min. 0, max. 6). The growth scores were comparable between PVL-positive and PVL-negative *S. aureus* isolates (D). However, the growth was significantly dependent on the competitor *P. aeruginosa* strain (E)

Due to the well-described co-detection of *P. aeruginosa* and *S. aureus* (irrespective of PVL), we performed a cross-streak assay with a *P. aeruginosa* isolate from this study (0093, co-isolated from a wound with a PVL-positive *S. aureus*) and a standard *P. aeruginosa* strain (ATCC 27,853). These were co-cultured with seven PVL-positive and seven PVL-negative *S. aureus* isolates from this study. The median *S. aureus* growth scores were comparable between PVL-positive and PVL-negative isolates (using both 0093 and ATCC 27,853 as competitors, 2.5 vs. 3, p = 0.87, Fig. 2). *S. aureus* growth strongly depended on the *P. aeruginosa* competitor: the median *S. aureus* growth score was significantly higher with the standard strain (ATCC 27,853) compared to the clinical isolate of this study (4 vs. 2, p = 0.0001, Fig. 2).

## Discussion

The main findings of our study were a frequent co-detection of *P. aeruginosa* and *S. aureus* and *K. pneumoniae* in chronic wounds.

The co-detection of *P. aeruginosa* and *S. aureus* in chronic infections (e.g. chronic wounds, cystic fibrosis) is well known and reflects a multi-layered interaction in which both species can inhibit (up-regulation of virulence factors) and promote each other (fitness gain, antimicrobial resistance, inhibition of opsonization) [19, 20]. To the best of our knowledge, the impact of PVL on the co-existence has not yet been investigated in detail. In vitro co-culture studies suggest that PVL confers a growth advantage for *S. aureus* as shown in a cross streak assay with *P. aeruginosa* wild type and *S. aureus lukS*-PV mutants [7]. PVL consists of the two subunits lukF-PV and lukS-PV, and the leukocidin is only active if both subunits are present [21]. If a competitive success of PVL-positive *S. aureus* over *P. aeruginosa* contributes to the widespread of PVL-positive isolates in Africa is part of current discussions. We did not detect an association of PVL with the absence of *P. aeruginosa* most likely due to the small sample size of PVL-*positive S. aureus*. Similarly, in the cross-streak assay (Fig. 2) we did not find any evidence that PVL-positive *S. aureus* inhibits the growth of *P. aeruginosa*. In contrast, the growth of *S. aureus* rather depended on the *P. aeruginosa* strain with a stronger inhibition of *S. aureus* by the clinical 0093 isolate compared to the ATCC 27,853 standard strain. This observation is in line with the study by Michelsen et al. who showed that inhibition of *S. aureus* by *P. aeruginosa* varies, and is strongest in less human-adapted isolates (e.g. early isolates of chronic infections) [18].

Our study has limitations. First, the long time span between sampling and culture certainly hampered the detection of fastidious bacteria. The low detection of anaerobic bacteria in our collection could suggest that we might underestimated fastidious genera. However, we rate the impact of this bias as low as a similar study that performed culture shortly after sampling in Ghana showed a comparable bacterial spectrum (i.e. predominance of *S. aureus*, *P. aeruginosa* or *Proteus*) in untreated Buruli ulcer cases [22]. In addition, (meta-) genomic profiling would have provided a more detailed picture of the wound microbiome [23]. Second, the aetiology of chronic wound infections in our study is not fully understood. It is possible that they may also be superinfected Buruli ulcers. Third, our findings on the microbial network represent a snapshot of the microbial community. If these communities are stable or if they dynamically change over time should be addressed in longitudinal observations.

## Conclusion

The culturome of chronic wounds in Sierra Leonean patients is divers and characterized by the co-detection of *P. aeruginosa*, *K. pneumoniae* and *S. aureus*.

## Electronic supplementary material

Below is the link to the electronic supplementary material.

Supplementary Material 1

## Data Availability

The datasets used and/or analysed during the current study are available from the corresponding author on reasonable request.
