# Correction: Network analysis of polymicrobial chronic wound infections in Masanga, Sierra Leone

**DOI:** 10.1186/s12879-023-08278-w

**Published:** 2023-05-25

**Authors:** Sarah Sandmann, Jonathan Vas Nunes, Martin P. Grobusch, Maxwell Sesay, Martin A. Kriegel, Julian Varghese, Frieder Schaumburg

**Affiliations:** 1 Institute of Medical Informatics, University of Münster, Münster, Germany; 2 Masanga Medical Research Unit (MMRU), Department of Infectious Diseases, Center of Tropical Medicine and Travel Medicine, Amsterdam University Medical Centers, Masanga, Amsterdam, Sierra Leone The Netherlands; 3 Section of Rheumatology and Clinical Immunology, Department of Medicine, University Hospital Münster, Münster, Germany; 4 Department of Translational Rheumatology and Immunology, Institute of Musculoskeletal Medicine, University of Münster, Münster, Germany; 5 Cells in Motion Interfaculty Centre, University of Münster, Münster, Germany; 6 Department of Immunobiology, Yale University School of Medicine, New Haven, USA

Correction: BMC Infect Dis 23, 250 (2023)

10.1186/s12879-023-08204-0

The original publication of this article [1] contained an incorrect title. The incorrect and correct title are shown in this correction article. The original article has been updated.

**Incorrect title**:

Research article network analysis of polymicrobial chronic wound infections in Masanga, Sierra Leone

**Correct title**:

Network analysis of polymicrobial chronic wound infections in Masanga, Sierra Leone.

The original article [1] has been updated.
