# Supplementary material for: Network analysis of polymicrobial chronic wound infections in Masanga, Sierra Leone
Source: BMC Infect Dis. 2023 Apr 18;23:250. doi: 10.1186/s12879-023-08204-0 (PMC10112320; doi:10.1186/s12879-023-08204-0)
Supplement: Supplementary file 1 — Supplementary Material 1 [file 12879_2023_8204_MOESM1_ESM.pdf]

Supplementary material

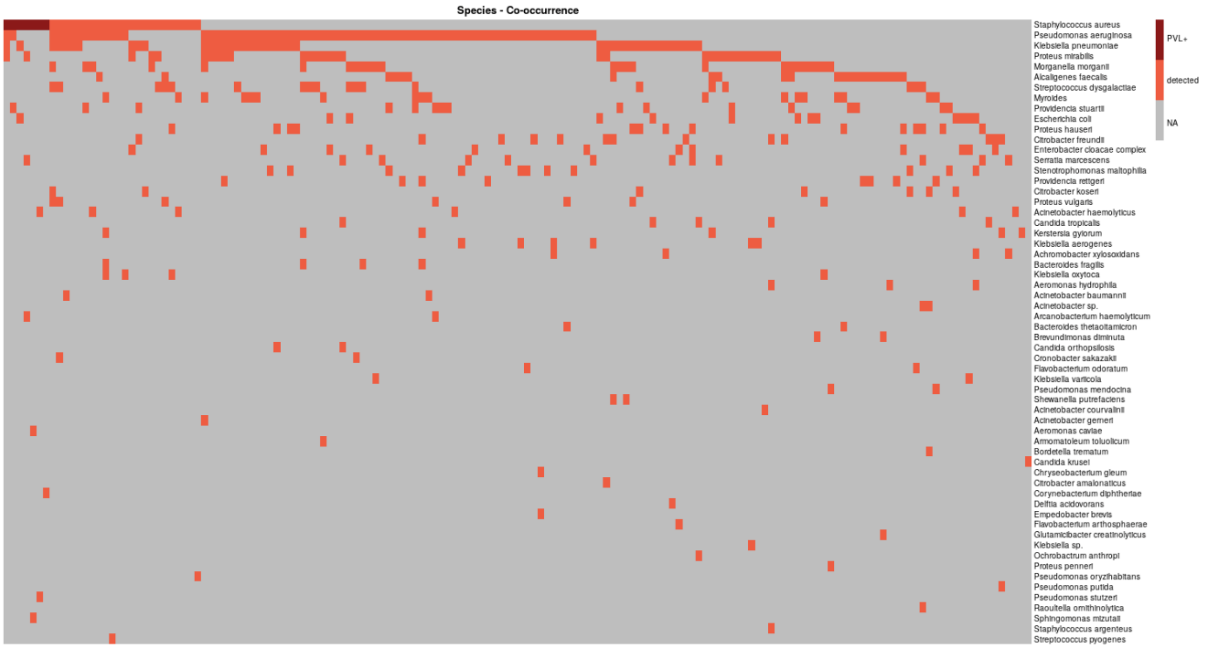

Figure S1: Heatmap visualizing co-occurring bacteria from chronic wound infections, Sierra Leone. Data is sorted by decreasing number of infections per species. For *S. aureus*, we differentiate between PVL-positive (dark red) and PVL-negative (light red) cases.
